# The Calcimimetic R568 Reduces Vascular Smooth Muscle Cell Calcification in Vitro Via ERK 1/2 Phosphorylation

**DOI:** 10.1155/ijne/2492846

**Published:** 2025-03-18

**Authors:** Jonas Engeßer, Philipp Gregor Albert, Matthias Scheuch, Norina Loth, Sylvia Stracke

**Affiliations:** ^1^Department of Internal Medicine A, Division of Nephrology, University Medicine Greifswald, Greifswald, Germany; ^2^Department of Internal Medicine A, Nephrology Research-Laboratory, University Medicine Greifswald, Greifswald, Germany

**Keywords:** apoptosis, calcimimetics, ERK 1/2, vascular calcification, vascular smooth muscle cells

## Abstract

**Background:** Vascular calcification (VC) is a common complication of chronic kidney disease, ultimately leading to high morbidity and cardiovascular mortality. In this study, we investigated the effects of the calcimimetic R568 in an in vitro model of human vascular smooth muscle cell (VSMC) calcification.

**Methods:** Human VSMCs were cultured under elevated calcium (2.4 mmol/L) and phosphate (2.7 mmol/L) concentrations. Calcification was analyzed using von Kossa staining and colorimetric calcium measurement. Intracellular signaling was examined via Western blot, and apoptosis was assessed by the TUNEL assay.

**Results:** Treatment with R568 significantly reduced VC over the 9-day treatment period. R568 treatment led to increased phosphorylation of extracellular signal-regulated kinase (ERK 1/2) compared to the control group. Calcimimetic treatment was also associated with a reduction in apoptosis. Blocking ERK 1/2 phosphorylation completely abolished the inhibitory effects of R568 on VC.

**Conclusion:** Our study provides new insights into the mechanism of action of calcimimetics during VC and highlights the importance of ERK 1/2 signaling in this process.

## 1. Introduction

Patients suffering from chronic kidney disease (CKD), particularly end-stage renal disease (ESRD), exhibit markedly increased mortality, primarily due to major cardiovascular events [1, 2]. A key driver of these cardiovascular events is vascular calcification (VC) [3]. Numerous studies have shown that approximately 50%–90% of patients undergoing dialysis develop VC [4]. Abnormal serum levels of calcium and phosphate are key pathophysiological mechanisms involved. Recent investigations have shown that VC is not a passive process where calcification occurs through simple deposition of calcium and phosphate but rather an active, cell-mediated process involving vascular smooth muscle cells (VSMCs) within the tunica media of arterial vessels. Following high calcium and/or phosphate exposure, calcification-inhibiting proteins, such as matrix Gla protein and fetuin-A, become depleted, leading VSMCs to release matrix vesicles without sufficient calcification inhibitors that cause extensive extracellular calcification [5, 6]. Once this mechanism is also exhausted, an increase in apoptotic VSMCs is observed, which dramatically enhances VC [7].

Over recent years, various calcification inhibitors have been identified and studied. Cinacalcet, an allosteric modulator of the calcium-sensing receptor, has garnered particular interest due to its effective treatment of secondary hyperparathyroidism in CKD-mineral and bone disorder [8, 9] as well as its direct action on VSMCs, reducing VC in vitro and in murine models [10, 11]. However, in the clinical setting, calcimimetics have shown differing results. Smaller studies have indicated a reduction in hospitalization due to cardiovascular events [12, 13], whereas the large EVOLVE study detected only a nonsignificant reduction in major cardiovascular events [14]. Given the discrepancy between the potent effects of calcimimetics observed in preclinical studies and their outcomes in the clinical setting, the aim of this study was to better characterize the mechanisms of action of the calcimimetic R568 in the context of VC.

## 2. Materials and Methods

### 2.1. Cell Culture and Calcification Assay

For all experiments, human coronary artery smooth muscle cells (Lonza, USA) were used within passages 5 to 8 to avoid replicative senescence [15]. Human VSMCs (70,000 cells per 12-well plate, 120,000 cells per 6-well plate, and 5000 cells per 8-chamber slide) were cultured in Medium 231 (ThermoFischer, Germany), supplemented with Smooth Muscle Growth Supplement 20x (ThermoFischer, Germany), and 5% penicillin/streptomycin (ThermoFischer, Germany) at 37°C and 5% CO_2_ until growing to confluency. Confluency was defined as covering > 95% of the culture dish/well plate with the typical “layer over layer pattern” of VSMCs representing the tunica media of arterial vessels. The medium was changed every other day.

### 2.2. Induction of VC in vitro

Calcification was induced using a calcification medium (CM) containing increased levels of calcium and phosphate to a final concentration of 2.4 mmol/L calcium and 2.7 mmol/L phosphate in addition to the growth medium in the presence or absence of R568 (0.1 μM, Tocris Biosciences, Cat. no. 3815). The concentration of R568 was chosen based on previous reports using a similar model [10]. The medium was changed every other day. For blocking ERK signaling, PD98059 (Cell Signaling Technology Europe, Cat. no. 9900S) in a final concentration of 10 μM was used.

### 2.3. Quantitative and Qualitative Analysis of Calcification

Quantification of VSMC calcification was performed using the O-Cresolphthalein-Complexon Assay. After 5, 7, or 9 days of VSMC cultivation in CM with or without R568, cells were washed with PBS and incubated with 0.6 M HCl at room temperature for 24 h. The supernatant was measured with a photometer and normalized against total protein concentration. Total protein concentration was measured using the mBCA-Kit (Thermo Fisher, Germany) according to the manufacturer's guide. For the ERK 1/2 inhibition experiment, PD98059 (Cell Signaling) at a concentration of 10 μM was added to R568.

Visualization of calcification was performed by using von Kossa staining. After 5, 7, or 9 days of VSMC cultivation in CM with or without R568, cells were washed and fixated with 4% PFA for 30 min at room temperature. After washing with PBS and deionized water, staining was performed by using AgNO_3_ (5% solution, Carl Roth, Germany, Cat. no. 9370.4) followed by exposure to UV light for 45 min. Excessive AgNO_3_ was removed by washing with deionized water and incubation with Na_2_S_2_O_3_ (5% solution, Carl Roth, Germany, Cat. no. HN25.1) for 5 min. Images were taken using an inverted phase contrast microscope (Zeiss Primo Star).

### 2.4. Western Blot Analysis

For analysis of ERK 1/2, signaling cells were treated with serum and antibiotic-free M231 medium for 12 h. Afterward, cells were treated for 30 min with either R568 in CM or CM alone. For analysis of osteogenic markers, cells were treated for 5, 7, or 9 days as described above. Cells were then washed with PBS. Ice-cold RIPA buffer with freshly added PMSF (0.1 M PMSF in isopropanol) and o-vanadate (ThermoFischer, Germany) was added to the cells for lysis. The cell suspension was transferred to a tube and treated with ultrasound for 10 s at 80% power. Protein concentration was measured via the bradford assay. Equal amounts of protein (20–30 μg) were loaded onto an SDS-polyacrylamid gel (10 or 12.5%) for electrophoresis, followed by a semidry blot onto nitrocellulose for 30 min at 25 V. Afterward, the membrane was blocked in 5% bovine serum albumin in TBS-T for 1 h at room temperature. Incubation with primary antibody against RUNX2 (#8486 Cell Signaling Technology, Europe), GAPDH (GE Healthcare UK), p44/42 MAPK (ERK 1/2) antibody (#9102 Cell Signaling Technology, Europe), and Phspho-p44/42 MAP (ERK 1/2) (Thr202/Tyr204) antibody (#4370 Cell Signaling Technology, Europe) was performed in TBS-T with 5% BSA at 4°C overnight. After washing with TBS-T, HRP-conjugated secondary antibodies (GE Healthcare, UK) were used, and the membrane was visualized with an ECL-Kit (WesternBright Cemilumineszenz, Biozym Scientific GmbH) according to the manufacturer's guide. Quantification of bands was performed by using a Vilber Lourmat Fusion Fx imager (Vilber Lourmat Smart Imaging).

### 2.5. Visualization of Apoptosis via the TUNEL Assay

VSMCs were cultivated in 8-chamber slides (5000 cells per chamber) and treated with CM w/or w/o R568 (0.1 μM, Tocris, UK) for 5, 7, and 9 days. Apoptosis was visualized by using the In Situ Cell Death Detection Kit, Fluorescein (Roche Diagnostics GmbH, Germany), according to the manufacturer's guide. DAPI staining was performed to visualize nuclei. Slides were covered in mounting media, and images were taken. Analysis was performed by a blinded investigator counting the percentages of apoptotic cells.

### 2.6. Statistical Analysis

Unless otherwise indicated, results are presented as mean ± standard error of the mean. Statistical analysis of data was performed using GraphPad Prism. After the D'Agostino–Pearson omnibus normality test, either Student's *t*-test or the Mann–Whitney test was used. For repeated measurements, an unpaired *t*-test with correction for multiple comparisons using the Holm–Sidak method was performed. A level of *p* < 0.05 was considered statistically significant.

## 3. Results

### 3.1. Calcimimetic Treatment Significantly Inhibits VC Over a 9-Day Course

**Figure 1:**
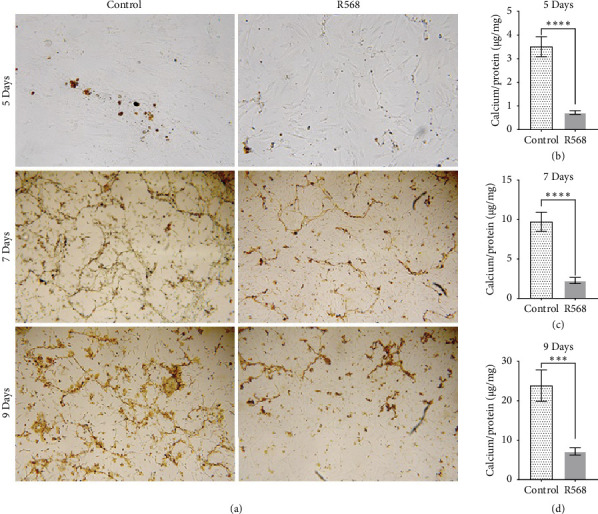
Calcimimetic treatment significantly prevents vascular calcification. (a) Visualization of VC through von kossa staining. Top row: 100x magnification and middle and lower row: 40x magnification. The control group showing increasing VC through the 9-day treatment beginning with small calcification nidi at day 5 and progressing to a honeycomb pattern at day 9. R568 showing a reduced severity of calcification. Pictures in (a) based on Engeßer [31] (b) quantification of calcium load through o-cresolphtalein-complexon assays show a significant reduction at 5 days of treatment with R568 compared to the control group (R568 0.721 ± 0.2828 *p* < 0.0001). (c) This is also present at 7 days (R568 2.281 ± 1.486 *p* < 0.0001). (d) And continues at day 9 (R568 7171 ± 3.632 *p*=0.0002).

To access the effects of R568 (0.1 μM) on VC, human VSMCs derived from coronary arteries were incubated in CM (Ca^2+^ 2.4 mmol/L and Pi 2.7 mmol/L) for up to 9 days. To identify VC in a qualitative manner, von Kossa staining was performed. For quantitative analysis, an O-Cresolphthaletin-Complexon assay was used. Incubation of VSMC in CM led to an increase in calcification of the VSMCs, starting with the formation of small calcification nidi on day 5 and then progressing to a honeycomb pattern (Figure 1(a)). In the quantitative analysis, R568 showed a high capacity in significant amelioration of vascular calcification over the 9-day treatment (Figures 1(a), 1(b), 1(c), and 1(d)), highlighting the potent effect of calcimimetics to reduce VC in vitro.

### 3.2. Coincubation of R568 With TGF-β1 Reduces R568-Induced ERK 1/2 Phosphorylation

**Figure 2:**
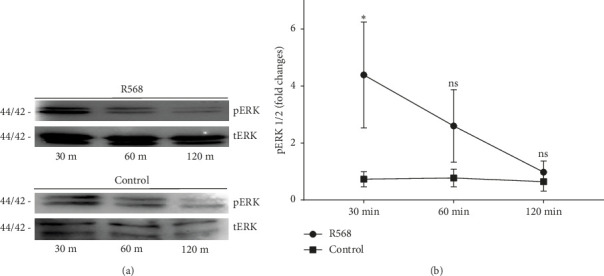
The calcimimetic R568 significantly induces ERK1/2 under procalcifying conditions. (a) Representative Western blot showing the strongest ERK phosphorylation at 30 min compared to the control group. Plots from (a) based on Engeßer [31] (b) quantification of western blot analysis visualizing the strongest ERK 1/2 phosphorylation at 30 min, *p*=0.0193, ns: nonsignificant (R568 4.3867 ± 3.222, control 0.73 ± 0.4603). (control = CM; R568 = CM + 0.1 mM R568).

To investigate the mechanisms through which R568 acts intracellularly, we took a closer look at the ERK 1/2 signaling pathway because calcimimetics are known to restore calcium-sensing receptor function in VSMCs under calcifying conditions. Before treatment, cells were kept on serum-starved medium to eliminate potential confounding agents. We established a time kinetic of ERK 1/2 phosphorylation, as ERK 1/2 phosphorylation is a rather fast-acting process. Activation was strongest after 30 min of R568 treatment and decreased over time (Figure 2(a)). At 30 min, R568 treatment was able to significantly increase ERK 1/2 phosphorylation compared to the control group (Figure 2(b)).

### 3.3. Blocking ERK 1/2 Signaling in VSMCs Abolishes the Inhibitory Effects of R568 on VC

**Figure 3:**
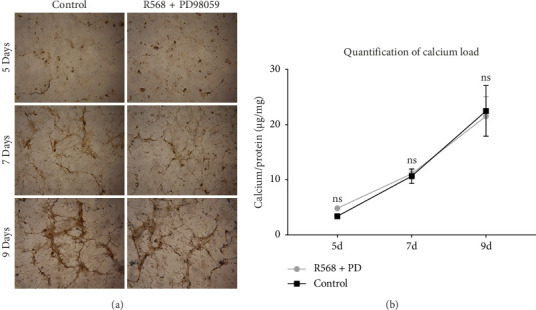
Blocking ERK 1/2 phosphorylation abolishes the inhibitory effects of R568 on vascular calcification. (a) Von kossa staining of VSMC under calcification conditions. Treatment of VSMC with PD98059 in addition to R568 showing a visually similar calcification severity compared to the control group. (b) The O-cresolphthalein-complexon assay showing that PD98059 completely eliminated the inhibitory effects of R568 on VC. ns, nonsignificant (control = CM; R568 = CM + 0.1 mM R568). Based on Engeßer [31].

To confirm our hypothesis that ERK 1/2 signaling is crucial for mediating the inhibitory effects of R568 on vascular calcification, we performed quantitative and qualitative calcium measurements in our in vitro model of VC while blocking ERK 1/2 signaling with the specific inhibitor PD98059 (10 μM). Von Kossa staining of the control group showed the same pattern as in Figure 1(a) with the beginning of calcification by forming nidi and then progressing to a honeycomb pattern. Coincubation of R568 with PD98059 completely abolished the inhibition of calcification of R568 on VC compared to the control group (Figure 3(a)), showing a near-identical calcification pattern. This could also be confirmed in the quantitative calcium measurement via the O-Cresolphthalein-Complexon assay, where coincubation of R568 and PD98059 produced a nearly identical progress of calcification (Figure 3(b)), showing the importance of ERK phosphorylation through calcimimetics.

### 3.4. Calcimimetic Treatment Reduces Apoptosis in Vitro

**Figure 4:**
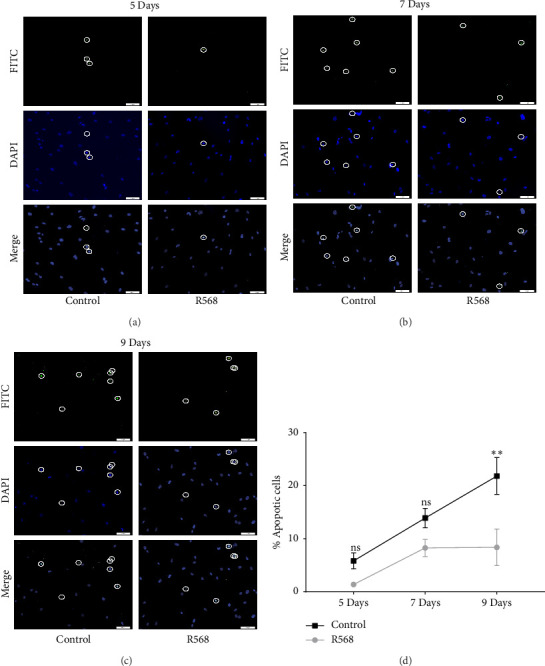
TUNEL assay revealing a reduction of apoptosis with R568 treatment. (a) The TUNEL assay at 5 days. The top row showing FIT-C positive apoptotic bodies, the middle row showing DAPI nuclei/DNA staining, and the lower row showing merge of the FIT-C and DAPI channels. Circles indicate apoptotic bodies. R568 shows reduced apoptosis rates compared to the control group. (b) The TUNEL assay at 7 days showing an increase in apoptosis compared to 5 days and still a reduction in apoptosis in the R568 treatment group. (c) The TUNEL assay at 9 days showing a further increase in apoptosis, while R568 still reduces apoptosis compared to the control group. Panels (a–c) based on Engeßer [31] (d) quantification of apoptotic cells showing an increase of apoptosis over time with a marked reduction of apoptosis in the R568 treatment group (*p*=0.0044, ns: nonsignificant) (control = CM; R568 = CM + 0.1 mM R568).

A key driver of VC is apoptosis. To examine the effects of R568 on apoptosis during VC, we conducted a transferase-mediated dUTP nick-end labeling (TUNEL) assay. VSMCs exposed to CM showed increasing rates of apoptosis throughout the 9-day treatment course, confirming the reduced cell numbers observed in the von Kossa staining for visualization of VC. Treatment with R568 resulted in decreased rates of apoptosis at all three time points (Figures 4(a), 4(b), 4(c)), showing a significant reduction at 9 days (Figure 4(d)), highlighting not only the calcification-inhibiting capacities of calcimimetics but also the ability to preserve cell survival, and thus potentially furthermore reducing vascular calcification.

## 4. Discussion

Over recent years, calcimimetics have garnered significant attention due to their ability to reduce serum parathyroid hormone levels in secondary hyperparathyroidism [16–18]. Additionally, calcimimetics exert direct effects on VSMCs via calcium-sensing receptor (CaSR) activation, thereby reducing VC in vitro and in murine models. Our study aligns with previous research, demonstrating the potent inhibition of VC by calcimimetics [10, 11, 19]. Prior studies have shown that a functional CaSR induces ERK 1/2 phosphorylation in VSMCs and mesenchymal cells [20, 21], so we were interested in whether calcimimetics preserve ERK signaling in VSMCs during VC and if this has an impact on the course of VC. Interestingly, the role of ERK in VC remains ambiguous. On one hand, previous data indicates that ERK activation is generally associated with increased cell survival via inhibition of proapoptotic proteins and induction of antiapoptotic proteins [22]. In the context of VC, ERK 1/2 phosphorylation has been shown to reduce VC by decreasing apoptosis [23–25]. On the other hand, ERK is proposed to promote VC through osteogenic transdifferentiation (OT) [26, 27]. OT is a process in which VSMCs acquire an osteoblast-like phenotype, a hallmark of VC. This process involves the upregulation of osteogenic transcription factors and mineralization markers. In our study, we observed that RUNX2, a key transcription factor and marker of OT that regulates the transcription of downstream genes involved in matrix mineralization, such as osteocalcin and bone sialoprotein, was expressed despite calcimimetic treatment (Supporting Figure 1). This suggests that treatment with R568 does not effectively prevent OT and supports previous findings that calcimimetics have only minimal or no impact on the expression of osteogenic markers in *in vitro* models of VC [10, 28, 29].

Our findings indicate that ERK 1/2 signaling is directly involved in preventing vascular calcification, as blocking ERK signaling completely abolished the effects of R568 on VC in vitro. Furthermore, R568 treatment was associated with reduced apoptosis in the context of VC at all time points of our study, supporting the idea that apoptosis is a major driver of VC and that calcimimetics restore ERK 1/2 signaling, ultimately reducing apoptosis.

Summarizing these findings, ERK activation is complex. On one hand, it can induce OT, which is thought to promote VC. On the other hand, it reduces vascular calcification by promoting VSMC survival. At this point, one could speculate that apoptosis is a stronger driver of VC than osteogenic transdifferentiation of VSMCs. Nevertheless, further investigations are necessary to elucidate the complex signaling pathways involved in VC.

There are certain limitations to this study. We used an in vitro model of human coronary VSMCs, which allowed us to test our hypothesis in the context of human samples with high clinical relevance, particularly considering ischemic cardiomyopathy as a key issue in the excessive mortality of ESRD patients. This is important because VSMCs from different sites have distinct origins and more than 3600 differentially expressed genes [30]. However, in vitro models are highly abstracted and cannot fully replicate the complexity of in vivo models and should be considered hypothesis-generating.

## 5. Conclusion

In conclusion, our results demonstrate that the calcimimetic R568 reduces vascular calcification through ERK 1/2 signaling and support the idea of targeting ERK 1/2 signaling in VC as a potential treatment strategy. Additional research is essential, as an effective treatment for VC is still not available.

## Data Availability

The data that support the findings of this study are available from the corresponding author upon reasonable request.

## Supporting Information

Supporting Figure 1: RUNX2 expression is present despite calcimimetic treatment, with representative Western blots showing that RUNX2 is expressed at all three time points of analysis (data from two independent experiments) based on Engeßer [31].
