# Supplementary figures and images for: The Calcimimetic R568 Reduces Vascular Smooth Muscle Cell Calcification in Vitro Via ERK 1/2 Phosphorylation
Source: Int J Nephrol. 2025 Mar 18;2025:2492846. doi: 10.1155/ijne/2492846 (PMC11936526; doi:10.1155/ijne/2492846)

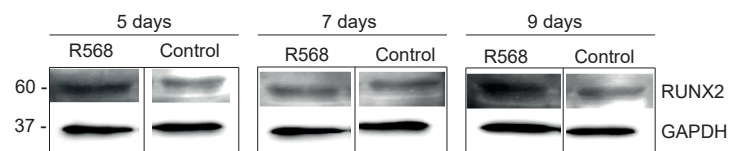

Supplement: Supporting Information — Additional supporting information can be found online in the Supporting Information section. [file 2492846.f1.pdf]
